# Implicit Social Attunement and Alcohol Use: The Effect of Peer Feedback on Willingness to Drink in Social Settings

**DOI:** 10.1007/s11469-024-01371-4

**Published:** 2024-08-12

**Authors:** Emese Kroon, Ran Zhang, Karis Colyer-Patel, Alix Weidema, Doğa Ünsal, Helle Larsen, Janna Cousijn

**Affiliations:** 1 Department of Psychology, University of Amsterdam, Amsterdam, The Netherlands; 2 Neuroscience of Addiction (NofA) Lab, Center for Substance Use and Addiction Research (CESAR), Department of Psychology, Education & Child Studies, Erasmus University Rotterdam, Rotterdam, The Netherlands

**Keywords:** Peers, Peer influence, Social attunement, Alcohol use, Age

## Abstract

Social context plays an important role in alcohol consumption. While most studies focus on explicit social drinking norms, this study aimed to (1) develop an implicit social attunement (ISA) task to experimentally assess how willingness to drink alcohol is affected by social alcohol drinking (SAD), social non-alcohol drinking (SNAD), and social non-drinking (SND) settings and peer feedback on willingness to drink in these settings, and (2) assess how ISA is associated with explicit social attunement, age, alcohol use and related problems. Participants (*N* = 506) aged 16–60 years completed the ISA task and questionnaires assessing alcohol use and alcohol use–related problems, age, and explicit social attunement online. Willingness to drink was highest in the SAD setting (SAD: *M*(*SD*) = 5.70 (2.68); SNAD: *M*(*SD*) = 4.03 (2.20); SND: *M*(*SD*) = 2.02 (1.30)) and—regardless of social setting**—**lower peer willingness to drink induced higher ISA than higher peer willingness to drink (SAD: *t*(325) =  − 2.929, *p* = .035; SNAD: *t*(325) =  − 2.888 *p* = .036; SND: *t*(325) =  − 6.764, *p* < .001). Higher ISA to higher peer willingness in the SAD (*r* = .15, *p* = .001) and SNAD (*r* = .11, *p* = .011) settings was associated with higher alcohol use and related problems, while higher ISA to lower peer willingness in the SND setting was associated with lower alcohol use and related problems (*r* =  − .18, *p* = .002) and recent alcohol use (standard drinks: *r* =  − .14, *p* = .011; binge drinking days: *r* =  − .16, *p* = .005). Explicit social attunement, but not ISA, mediated the association between lower age and higher alcohol use and related problems (*b* =  − .013, *p* = .009). Results indicate that peer feedback can be a protective or risk factor for alcohol use, depending on the social setting. Future studies are needed to elucidate differences between implicit and explicit social attunement behaviors in their associations with age and alcohol use and related problems.

**Supplementary Information:**

The online version contains supplementary material available at 10.1007/s11469-024-01371-4.

Alcohol use in social settings is very common across age groups (e.g., Mustonen et al., 2014) and stronger social motives for use have been associated with higher frequency and quantity of alcohol consumption and alcohol use–related problems (Bresin & Mekawi, 2021). Peer drinking is known to be associated with higher alcohol use (Scholte et al., 2008), especially during adolescence (Huang et al., 2014), potentially due to heightened social reward sensitivity (Beard et al., 2022; Foulkes & Blakemore, 2016). However, most evidence of peer influence on alcohol use–related behaviors relies on self-report measures of explicit social norms. Hence, we know relatively little of the potential implicit nature of peer influence on alcohol consumption. Furthermore, it is unclear how contextual factors, age, and social attunement—individual tendencies to adapt to and harmonize with one’s social environment—might be associated with peer influence on willingness to drink.

Alcohol use is usually initiated during adolescence and peaks during early adulthood (Johnston et al., 1975; Lee et al., 2018). Social learning is crucial during this developmental period in which parental influence reduces while peer influence increases (Marshal & Chassin, 2000; Sebastian et al., 2008). However, peer influence on alcohol use remains prominent in adulthood: Rosenquist et al. (2010) showed that individuals with a heavy drinking social network are more likely to drink heavily themselves, and that the individuals closest to them have the largest influence on one’s drinking behavior. In line with this, decades of research have shown that both descriptive and injunctive peer norms, especially from proximal peers, are associated with heaviness of alcohol use (e.g., Halim et al., 2012; Voogt et al., 2013). For example, Leung et al. (2014) showed that adolescents with heavy drinking peers are more likely to heavily drink themselves due to peer influence and peer group selection. While solitary drinking might pose a higher risk for the development of an alcohol use disorder (AUD; e.g., Creswell, 2021; Skrzynski & Creswell, 2020), Creswell (2021) showed that drinking in social settings increases immediate health risks through the consumption of larger amounts of alcohol. For example, a recent ecological momentary assessment study by O’Donnell et al. (2019) showed that young adults consumed larger amounts of alcohol in social situations where others consumed alcohol. Furthermore, several experimental studies also showed that young adults adapt their drinking behavior to the drinking of other individuals (Larsen et al., 2009, 2010, 2012) and drink more in group settings (Kuendig & Kuntsche, 2012).

Peer influence on drinking can take a variety of forms. Peer pressure in which individuals conform with the group norm to avoid negative consequences is common (e.g., O’Donnell et al., 2019), but more positive reinforcement motives, like social attunement, might also play a role (Cousijn et al., 2018). Social attunement refers to an individual’s tendencies to adapt to and harmonize with one’s social environment—in the absence of explicit peer pressure—to maximize positive social feedback. Hence, social attunement could play a central role in reducing alcohol use when maturing (Chassin et al., 2004; Vergés et al., 2013): as reduced reward sensitivity and increased behavioral control only account for part of the reduction in alcohol use when maturing (Heyman, 2009), changing group norms and peer influence might be crucial in this process (Dawson et al., 2006; Lee et al., 2015). Social attunement is a form of social learning that is hypothesized to play an important role in both adolescent risk for excessive use and resilience to persistent heavy use in adulthood (Cousijn et al., 2018). While one’s tendency to attune with the social environment might result in excessive use during adolescence when the social value of excessive drinking can be relatively high, this same tendency might result in a reduction of drinking in adulthood by attuning to the new socially devaluated alcohol standards (e.g., due to new responsibilities; Hajema & Knibbe, 1998).

Recently, Kroon et al. (2022) developed a questionnaire to assess explicit self-reported social attunement and assessed the role of social attunement in alcohol use. Results showed that explicit social attunement is associated with alcohol use and related problems, specifically in youngsters (around 16 to 20 years old) with heavy drinking peers. However, it is likely that not all social attunement is explicit: we regularly adjust our behaviors to the social environment without noticing or being able to report on it. Hence, implicit measures of social attunement are crucial to explore the role of social attunement in alcohol use.

As tasks to assess implicit social attunement are currently lacking, we aimed to develop a task to experimentally assess the effects of different social settings (i.e., alcohol drinking, non-alcohol drinking, or no drinking) on willingness to drink, and whether peer feedback—either indicating higher or lower willingness to drink—results in implicit social attunement in the direction of the peers. We expect that implicit social attunement of one’s willingness to drink alcohol is higher in social settings in which other individuals are drinking alcohol, compared to situations with non-alcoholic drinks or no drinks. Furthermore, we expect implicit social attunement to depend on the direction of peer feedback: reducing willingness after lower drinking feedback, increasing willingness after higher drinking feedback, while not changing willingness when peer feedback matches one’s own willingness.

Second, we aimed to evaluate how implicit social attunement, explicit social attunement, and age are associated with alcohol use and whether implicit and/or explicit social attunement might mediate the association between age and alcohol use. Focusing on the task outcomes, we expect higher implicit social attunement when peers have a lower willingness to drink to be associated with lower alcohol use, while we expect higher implicit social attunement when peers have a higher willingness to drink to be associated with higher alcohol use. Also, we expect implicit social attunement to be positively associated with explicit social attunement and expect younger individuals to show higher implicit and explicit social attunement and to report higher alcohol use than their adult counterparts. Furthermore, we expect implicit and explicit social attunement to mediate the association between age and alcohol use.

This study proposes a novel task to measure implicit social attunement complementing the existing measure of explicit social attunement. This will enable us to assess the role of implicit and explicit social processes in alcohol use and how these differ depending on age.

## Methods

### Sample and Procedure

A total of 506 participants aged 16–60 participated in the study, to reach sufficient power for the most complex analyses (i.e., mediation models; Sim et al., 2022). The collected data was part of a larger online study including multiple cognitive tasks and questionnaires. Participants were recruited via social media (i.e., WhatsApp, Instagram, Facebook), in-person flyers (Amsterdam and Rotterdam region), and the Erasmus University Rotterdam’s student participant pool. The study advertisement was targeting individuals that use cannabis and/or alcohol, but no inclusion or exclusion criteria aside from age (16–65 years old) and proficiency in English or Dutch applied. Participants were fully informed, had the opportunity to contact the researchers, and signed informed consent before participation. The 1-h online session included two blocks of questionnaires, separated by several experimental tasks, that participants could complete in English or in Dutch depending on their preference. At the end of the study, participants were pointed towards potential ways to seek help for mental health or substance use problems. As compensation, participants could enter a raffle in which one in five participants received a ten-euro gift card. Study protocols were approved by the Ethics Review Committee of Erasmus University Rotterdam (ETH2122-0311) to ensure confidentiality, data protection, and informed consent.

### Measures

#### Implicit Social Attunement Task

The Implicit Social Attunement Task (ISAT; inspired by Izuma & Adolphs, 2013) was developed to measure implicit social attunement (ISA) to peer feedback in different imagined social situations. In this version, social attunement is operationalized as the extent to which participants adjust their initial willingness to drink in the direction of the peer feedback, controlled for average adjustments made when no feedback is provided.

In the first block, participants were presented with 45 images of social situations (featuring two or more men and/or women) in a fixed order, including 15 images for each of the three social situations: Social Alcoholic Drink (SAD; drinking beer), Social Non-Alcoholic Drink (SNAD; drinking soda), Social Non-Drink (SND; no drinking). Participants were instructed to imagine being in the presented situation and asked to indicate their willingness to drink alcohol in this situation on a scale from 1 to 10 (1 = no willingness, 10 = strong willingness), enabling a large range of responses while using a common grading scale (i.e., Dutch grading system) which has previously been used and validated in similar studies (e.g., Larsen et al., 2022). Immediately after their response, they were shown what they were told was the average willingness to drink that their peers (a group of similar age) had indicated for this situation when they completed the task earlier. This “peer feedback” was not real: feedback was semi-randomly generated based on the participants’ response. Peers responded identically to the participants in 2 trials (fixed trials identical for all participants). In the other 43 trials, the chance of receiving “higher” willingness feedback (peer rated higher than participant) or “lower” willingness feedback (peer rated lower than participant) was 50%. In all trials with non-identical feedback, there was a 70% chance that the difference between participants’ and peer response was large (2 to 3 points) and a 30% chance that the difference was small (1 point). After the first block, a working memory task was performed, creating an 11-min delay. In the second block, all pictures were shown again in the same order and participants were asked to rate their willingness to drink in these situations again, without seeing peer feedback.

#### Questionnaires

Participants completed single-item questions assessing age (in years), gender (male/female/other), nationality (Dutch/Non-Dutch), highest completed education (recoded to four categories based on the Dutch high school (different levels preparing for different higher education levels), and higher education system: (1) less than high school, (2) (pre-) vocational/(pre-) college, (3) (pre-) university, (4) other, age of onset of alcohol use (in years), self-reported (estimated) number of days of binge drinking (> 4 drinks on a single occasion; Courtney & Polich, 2009) during the last year, and self-reported (estimated) lifetime drug use (excluding alcohol, cigarettes, and cannabis). The Alcohol Use Disorder Identification Test (AUDIT; Hildebrand & Noteborn, 2015: *α* = 0.92, Saunders et al., 1993) was used to assess alcohol use (AUDIT-C: items 1–3) and related problems (AUDIT-P: items 4–10), with full AUDIT (items 1–10) scores over 7 reflecting problematic alcohol use. Furthermore, participants completed a timeline follow-back (TLFB; Robinson et al., 2014) calendar assessing last 14-day use of alcohol (number of standard drinks, number of drinking days, number of binge drinking days), cigarettes (number of cigarettes), and cannabis (grams) to assess recent use. The social attunement questionnaire (SAQ; Kroon et al., 2022: *α* = 0.75) was used to assess explicit social attunement. General mental health was assessed using the DSM-5 Cross Cutting Symptoms scale (American Psychiatric Association, 2013; Doss & Lowmaster, 2022: *α* = 0.96) and general executive functioning was assessed using a short 6-item self-report questionnaire for online assessment (Buchanan et al., 2010: *α* = 0.79).

### Data Analysis

First, to assess the effects of social setting and feedback type on willingness to drink and to evaluate the psychometric properties of the novel ISAT task, internal consistency of the three social conditions was assessed (Cronbach’s alpha) and a repeated measures ANOVA was conducted to compare willingness to drink in block 1 across the three social conditions (SAD, SNAD, SND). Sample *t*-tests were used to assess the presence of a change in willingness to drink (different from 0) in different social situations (SAD, SNAD, SND) for different feedback types (high, low, same).

Second, to assess the role of feedback type and social setting in implicit social attunement (ISA), ISA scores—controlling for change on same feedback trials—were calculated per participant to quantify individual implicit social attunement when provided with higher willingness to drink feedback (ISA +  = difference score on positive trials − difference scores on same trials) and when provided with lower willingness to drink feedback (ISA −  =  − 1*difference score on negative trials − difference score on same trials). Separate scores were calculated for all social settings (SAD, SNAD, and SND). Repeated measures ANOVAs were conducted to assess the effects of social setting (SAD, SNAD, SND), feedback type (+ , −), and their interaction on ISA.

Third, to assess how ISA scores were associated with explicit social attunement, age, and alcohol use, Spearman’s correlations between ISA + and ISA − scores per social setting, SAQ scores, age, and measures of alcohol use (AUDIT and TLFB outcomes) were calculated. Then, exploratory mediation analyses were conducted to assess whether SAQ and/or ISA scores mediated the association between age and alcohol use measures. Analyses were conducted using the most recent versions of R (version 4.2.2 in RStudio version 2022.12.0) and JASP (version 0.16.4).

## Results

### Sample Characteristics

The majority of the participants were non-Dutch (66.60%), highly educated (94.47% completed (pre-)university education), and identified as female (60.08%), with an average age of 29.90 (*SD* = 11.85) years old (Table 1). Most participants reported lifetime alcohol use (93.87%), on average reporting low-risk alcohol consumption in the last year (AUDIT < 8) (Saunders et al., 1993) and drinking about 1 in 3 days during the last 2 weeks (Table 1).

**Table 1:** Sample characteristics

Categorical outcomes	Description		*N*	Percentage
Gender	Male/female/other		188/304/14	37.15/60.08/2.77
Nationality	Dutch/other		169/337	33.40/66.60
Education	Less than high school/(pre-) vocational or college/(pre-) university/other		6/16/478/6	1.19/3.16/94.47/1.19
Lifetime alcohol use	Yes/no		475/31	93.87/6.13
Lifetime cigarette use	Yes/no		327/179	64.63/35.38
Lifetime cannabis use	Yes/no		311/195	61.46/38.54
Lifetime other drug use^a^	Yes/no		300/206	59.29/40.71
High risk alcohol use (AUDIT > 7)	Yes/no		187/319	36.96/63.04
**Continuous outcomes**	**Cronbach’s alpha**	**M (SD)**	***N***	**Range**
Age	–	29.90 (11.85)	506	16–60
Mental health (DSM-5 CCS)	.93	14.19 (12.07)	506	0–74
Executive functioning	.88	11.61 (4.36)	506	6–24
Explicit social attunement (SAQ)	.81	43.13 (10.27)	506	11–74
Alcohol use and related problems (AUDIT – total)	.84	6.84 (5.63)	506	0–34
Alcohol use (AUDIT – items 1–3)	.75	3.90 (2.47)	506	0–12
Alcohol use related problems (AUDIT – items 4–10)	.78	2.97 (3.74)	506	0–24
Age of onset alcohol use	–	15.24 (2.85)	475	3–40
Binge drinking occasions last year	–	21.73 (40.43)	473	0–364
Last 14-day alcohol use (TLFB – standard drinks)	–	12.48 (17.00)	506	0–121
Last 14-day alcohol use (TLFB –drinking days)	–	3.52 (3.71)	506	0–14
Last 14-day alcohol use (TLFB – binge days)	–	0.88 (1.75)	506	0–14
Last 14-day cannabis use (TLFB – grams)	–	0.88 (3.86)	506	0–62
Last 14-day cigarette use (TLFB – cigarettes)	–	28.96 (71.92)	505	0–700

Note. ^a^including all drugs except alcohol, tobacco, and cannabis. *AUDIT*, alcohol use disorder identification test; *DSM-5 CCS*, DSM-5 Cross Cutting Symptoms Measure (higher scores indicate more mental health problems); *TLFB*, timeline follow-back; *SAQ*, social attunement questionnaire

### Willingness and Change in Willingness to Drink

**Fig. 1:**
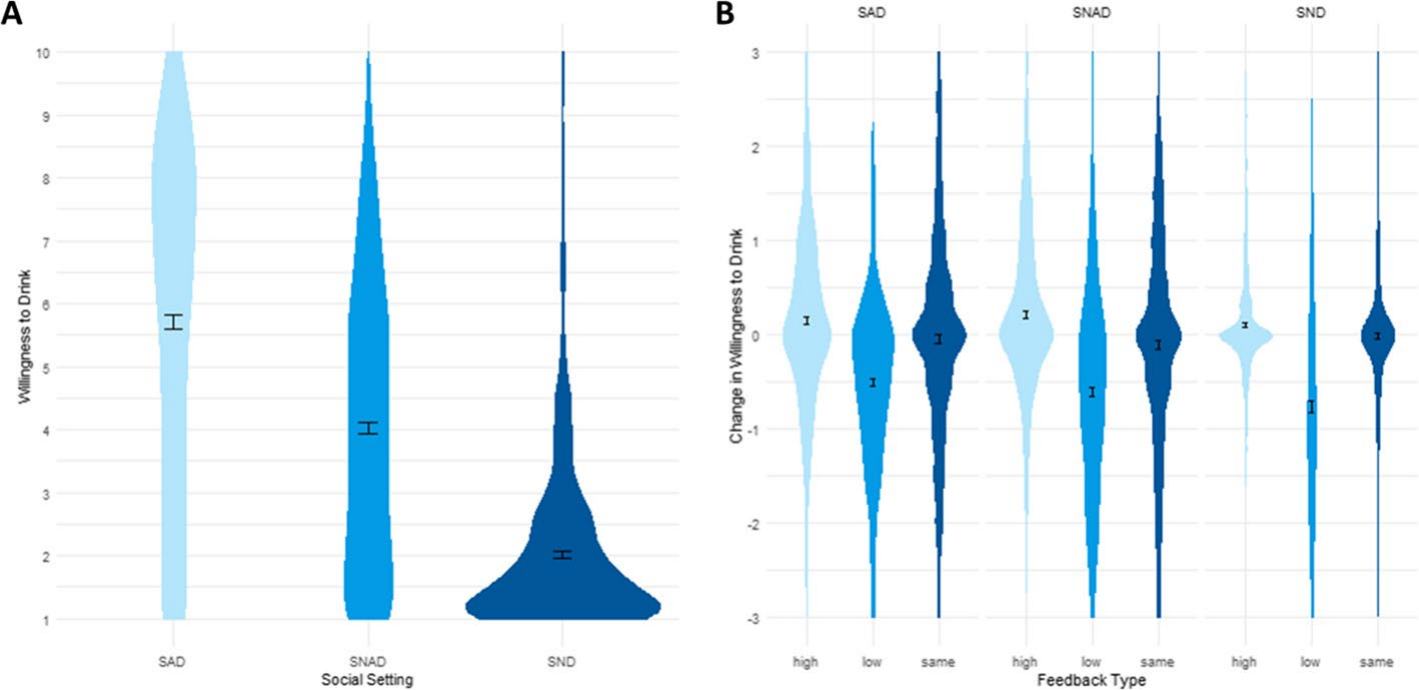
Willingness to drink per social setting (**A**) and changes in willingness to drink per social setting and feedback type (**B**). SAD, social alcohol drink; SNAD, social non-alcohol drink; SND, social non-drink; high: peer feedback indicating higher willingness to drink, low: peer feedback indicating lower willingness to drink, same: peer feedback identical to own willingness to drink. Error bars represent mean and SE

Social setting (SAD: *α* = 0.98, SNAD: *α* = 0.97, SND: *α* = 0.92) did significantly affect willingness to drink in block 1 (*F*(1.42, 802.84) = 989.69, *p* < 0.001, η_general_^2^ = 0.33; Fig. 1A). Holm’s corrected post hoc comparisons showed that there were significant differences in willingness to drink between all conditions (all *p* < 0.001), with willingness to drink being highest in the SAD setting and lowest in the SND setting (SAD: *M*(*SD*) = 5.70 (2.68); SNAD: *M*(*SD*) = 4.03 (2.20); SND: *M*(*SD*) = 2.02 (1.30); Table S1). Willingness to drink changed in the direction of the peer feedback (all *p*’s > 0.001, see Fig. 1B and Table S1), while no change in willingness was observed when presented with peer feedback indicating identical willingness to drink (lowest *p* = 0.143).

### Implicit Social Attunement: The Effect of Social Setting and Feedback Type

**Fig. 2:**
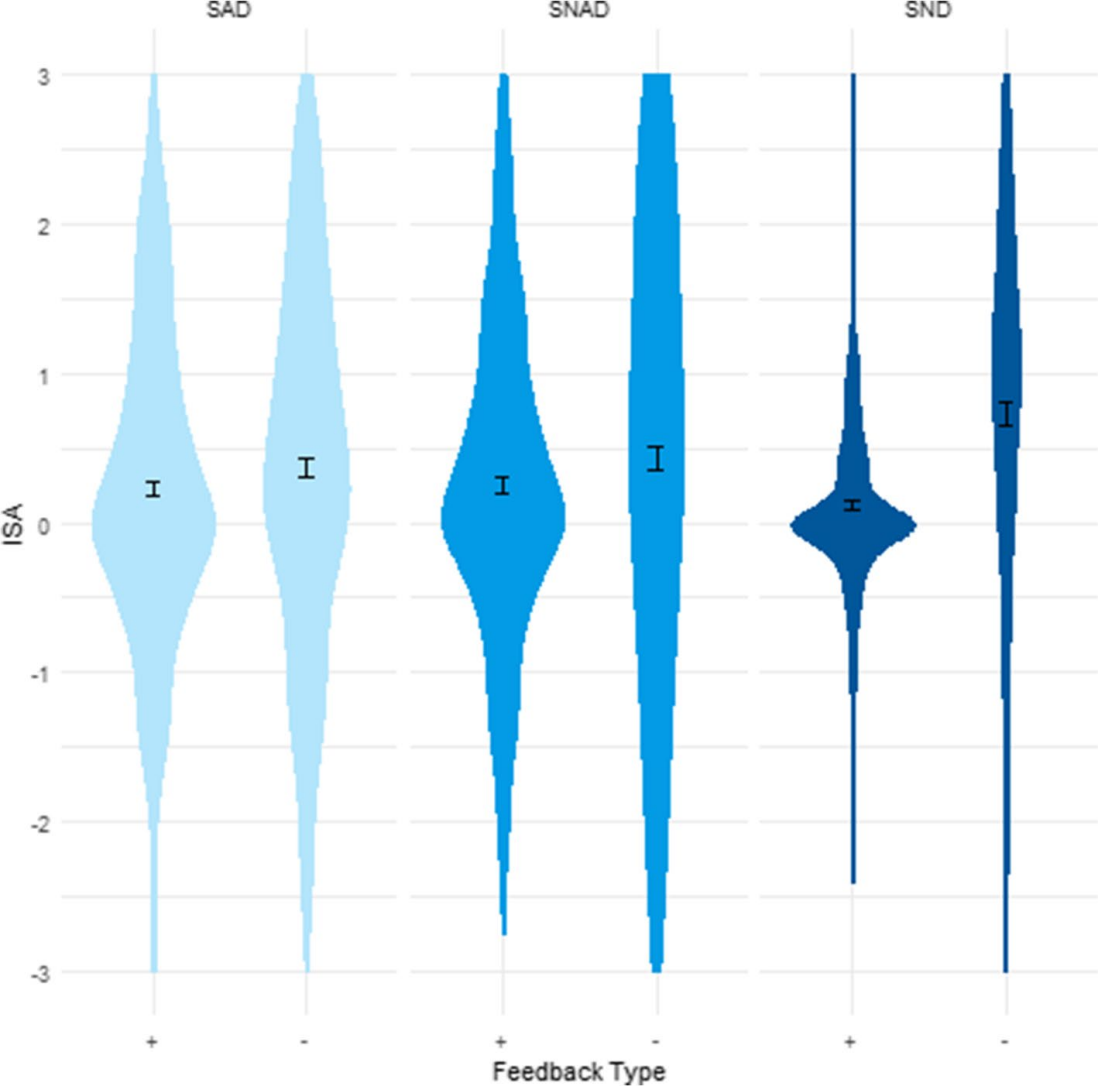
The effects of social setting and feedback type on implicit social attunement (ISA). SAD, social alcohol drink; SNAD, social non-alcohol drink; SND, social non-drink; + : peer feedback indicating higher willingness to drink, -: peer feedback indicating lower willingness to drink. ISA + and ISA − scores are corrected for change on same feedback trials. Error bars represent mean and SE

Results showed a significant interaction between feedback type and social setting in their effect on ISA (*F*(1.75, 568.53) = 6.846, *p* = 0.002, η_general_^2^ = 0.003; Greenhouse–Geisser correction applied) (Fig. 2). Holm’ corrected post hoc comparisons showed that there were no differences between social settings in ISA + scores (lowest *p* = 0.780) or ISA − scores (lowest *p* = 0.302; Table S2). Within social setting, feedback type did have an effect: ISA − scores were higher than ISA + scores in the SAD (*t*(325) =  − 2.929, *p* = 0.035, *d* = 0.325), SNAD (*t*(325) =  − 2.888, *p* = 0.036, *d* = 0.320), and SND (*t*(325) =  − 6.764, *p* < 0.001, *d* = 0.750) condition. There were differences between the ISA + and ISA − scores across all social settings, except for the comparisons between the SAD and SNAD conditions (SAD ISA + vs. SNAD ISA − : *t*(325) =  − 2.449 *p* = 0.116, *d* = 0.272; SNAD ISA + vs. SAD ISA − : *t*(325) =  − 1.940, *p* = 0.316, *d* = 0.215).

### Implicit Social Attunement: Associations with Explicit Social Attunement, Age, and Alcohol Use

**Fig. 3:**
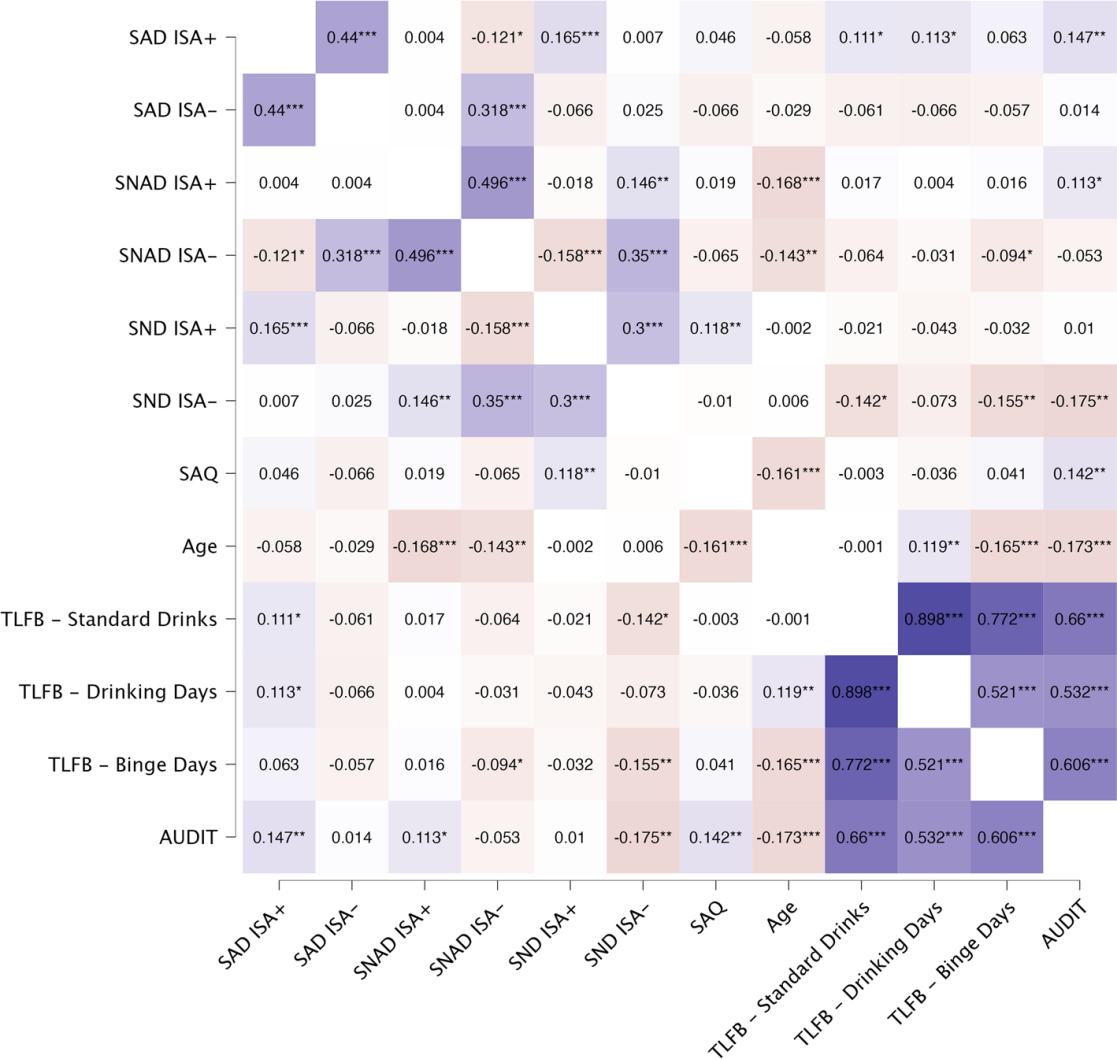
Correlations between implicit social attunement, explicit social attunement, age, and alcohol use. Positive correlations highlighted in purple and negative correlations highlighted in red, with darker colors presenting stronger correlations. SAD, social alcohol drink; SNAD, social non-alcohol drink; SND, social non-drink; ISA + , implicit social attunement to peer feedback indicating higher willingness to drink, controlled for same feedback trials responses; ISA − , implicit social attunement to peer feedback indicating lower willingness to drink, controlled for same feedback trials responses; AUDIT, alcohol use disorder identification test; TLFB, timeline follow-back. SAQ, social attunement questionnaire scores of explicit social attunement. Significance levels: * *p* < .05, ** *p* < .01, *** *p* < .001

Full AUDIT scores were positively correlated with ISA + in SAD (*r* = 0.15, *p* = 0.001) and SNAD (*r* = 0.11, *p* = 0.011) trials, but not SND trials (*p* = 0.83; Fig. 3; full overview in Table S3). However, TLFB scores were only positively correlated with ISA + in SAD trials (Standard drinks *r* = 0.11, *p* = 0.015; Drinking days *r* = 0.11, *p* = 0.013). Full AUDIT scores were negatively correlated with ISA − in SND trials only (full AUDIT: *r* =  − 0.18, *p* = 0.002). TLFB scores were negatively correlated with ISA − in the SND trials (Standard drinks: TLFB: *r* =  − 0.14, *p* = 0.011, Binge days: *r* =  − 0.16, *p* = 0.005) and SNAD trials (Binge days: *r* =  − 0.094, *p* = 0.049). There was a strong positive correlation between all TLFB outcomes and full AUDIT scores (Standard drinks: *r* = 0.66, *p* < 0.001; Drinking days: *r* = 0.53, *p* < 0.001; Binge days: *r* = 0.61, *p* < 0.001). Full AUDIT scores (*r* =  − 0.18, *p* < 0.001) and binge days (*r* =  − 0.17, *p* < 0.001) correlated negatively with age, while there was a positive correlation between age and drinking days (*r* = 0.12, *p* = 0.007). SAQ scores were positively correlated with full AUDIT scores (*r* = 0.14, *p* = 0.001), but not with any TLFB outcome. SAQ scores were positively correlated with ISA + SND trials (*r* = 0.18, *p* = 0.008), but not SNAD (*p* = 0.866) or SAD (*p* = 0.314) trials. SAQ scores were not associated with ISA − in any condition (lowest *p* = 0.174). Furthermore, SAQ scores were negatively associated with age (*r* =  − 0.17, *p* < 0.001).

### The Mediating Role of Implicit and Explicit Social Attunement

**Fig. 4:**
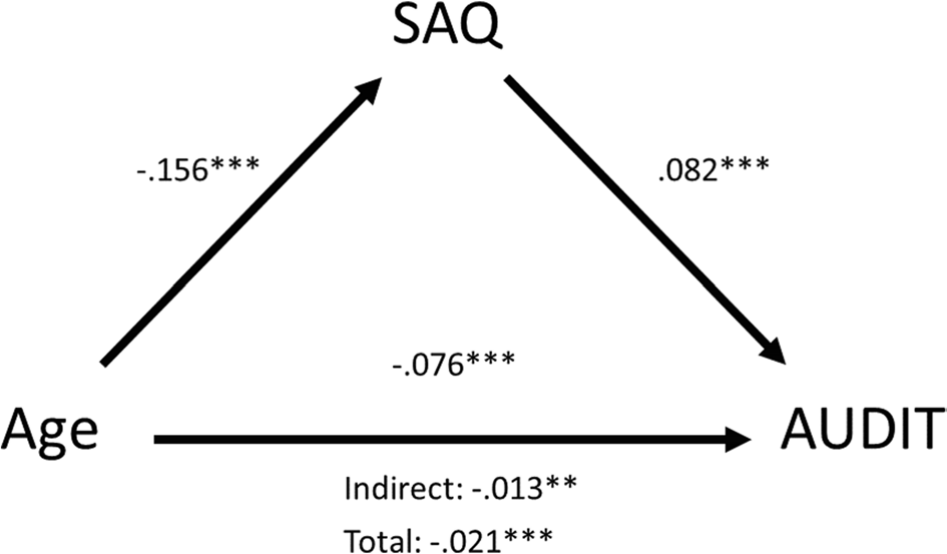
Mediation results: SAQ mediates the association between age and AUDIT scores. *** *p* < .001, ** *p* < .01

Whereas none of the ISA scores mediated the association between age and full AUDIT scores (Table S4) or age and any of the TLFB scores (Table S5–S7), SAQ scores did mediate the association between age and full AUDIT scores (*b* =  − 0.013, *SE* = 0.005, 95% *CI* = [− 0.023, − 0.005], *p* = 0.009; Fig. 4), but not the association between age and TLFB scores (Table S5–S7). Younger individuals showed higher explicit social attunement, which was related to higher full AUDIT scores. Sensitivity analyses showed that this effect was driven by individuals scoring above the full AUDIT cut-off for heavy use (AUDIT > 7; Table S8) and separate analyses of the alcohol use (AUDIT-C; items 1–3) and alcohol use–related problems (AUDIT-P; items 4–10) items of the AUDIT confirmed that the effects were guided by the alcohol use related problem items (Table S9).

## Discussion

Using the newly developed implicit social attunement task, we assessed the effects of social settings on willingness to drink and whether peer feedback would induce implicit social attunement towards peers. Willingness to drink was highest in social settings where alcohol was being consumed, indicating that perceived norms in social settings affect willingness to drink. Furthermore, task manipulations were effective: individuals adapted their willingness to drink in the direction of the peer feedback provided. Implicit social attunement did not differ between social settings, but social attunement was larger when peers indicated lower willingness to drink compared to higher willingness to drink. However, higher implicit social attunement to peers indicating higher willingness to drink in social drinking settings was associated with higher alcohol use and related problems. Furthermore, while implicit social attunement did not mediate the association between age and alcohol use measures, explicit social attunement did: younger individuals that reported higher explicit social attunement scored higher on alcohol use and related problems.

The implicit social attunement task showed excellent internal consistency (Tavakol & Dennick, 2011) in all social settings and willingness to drink changed in the direction of the peer feedback. As expected, willingness to drink was higher in the SAD condition than in the SNAD and SAD conditions, while willingness to drink was also higher in the SNAD than the SAD condition. This could indicate that seeing others drink increases willingness to drink regardless of the type of drink but that the effect is larger when the norm of drinking alcohol is present. This is in line with previous research showing that injunctive and descriptive peer norms (e.g., Halim et al., 2012; Voogt et al., 2013) and imitation of peer behavior (Larsen et al., 2009, 2010, 2012) affect people’s drinking behavior. Using this task, we were also able to distinguish the effects of different social settings within the same task. Interestingly, results indicate that people show lower willingness to drink when no alcohol is being consumed, indicating that peer influence and descriptive norms might also be important in reducing alcohol consumption.

Regardless of social setting, individuals did attune their willingness to drink in the direction of the peers. This effect is larger when provided with peer feedback indicating lower willingness to drink, also highlighting the potential importance of peer influence in cutting down alcohol use. This is in line with earlier research by Stevens-Watkins and Rostosky (2010) highlighting the potential protective factors of lower perceived peer substance use during adolescence on (binge) drinking in later years. Research should be extended, varying the group that provides feedback, to assess whether effects are similar when feedback is provided by proximal peers—usually expected to have greater influence (e.g., Salvy et al., 2014; Voogt et al., 2013)—parents, or health care professionals to assess generalizability and potential prevention effects. Unlike expected, social setting did not affect social attunement. We expected that the presented social setting would affect the perceived drinking norms, resulting in larger attunement when feedback indicating higher willingness to drink is provided in the SAD setting and when feedback indicating lower willingness to drink is provided in the SNAD and SND settings (Halim et al., 2012; Voogt et al., 2013). These results could be associated with the fact that the norm setting was implicit rather than often studied explicitly reported injunctive and descriptive norms (e.g., Voogt et al., 2013) and did not include a real-life drinking situation in which imitation might play a larger role (e.g., Larsen et al., 2012). However, these results also highlight the potential irrelevance of social setting in implicit peer influence and/or large individual differences in the relevance of social setting. It could well be that the behavior of peers and the social norm set by this behavior is more important than the specific social setting when it comes to alcohol use, but future studies should replicate these findings to confirm potential utility in intervention settings.

Correlational analyses showed that only in non-alcohol settings (SNAD and SND), implicit social attunement to lower peer willingness to drink was associated to less alcohol use and related problems. This may mean that those with less implicit social attunement in response to peers indicating lower willingness to drink in non-alcohol settings tend to drink more and experience more alcohol use–related problems. In contrast, only in the settings in which drinks were present (SAD and SNAD), implicit social attunement to higher peer willingness to drink was associated with more alcohol use and related problems. This indicates that those that attune less to peers with higher willingness to drink in drink settings consume less and experience less alcohol use–related problems. Although speculative, these results indicate that individuals who reduce their willingness to drink in social settings where peers are not drinking may be more resilient and less likely to develop alcohol-related problems. Conversely, those who increase their willingness to drink in social drinking settings where peers are willing to drink could be at higher risk. These results are in line with recent research showing that those with a higher resistance to peer influence show smaller associations between perceived peer drinking and own alcohol use (DiGuiseppi et al., 2018). Interestingly, this study did not find similar associations with actual peer drinking, indicating a need for assessing ecological validity of our task and how results relate to real-life drinking behavior. For example, studies should include measures of perceived peer drinking and actual peer drinking within the individuals’ proximal peer group to assess whether social attunement is involved in the association between real-life peer drinking experiences and real-life drinking behavior (Kroon et al., 2022).

Regarding the role of age, we found implicit social attunement in SNAD settings to be higher in younger individuals, indicating that in potentially ambiguous situations (no alcohol drinks, but people are having a drink) younger adults might have a higher tendency to attune to peer feedback regardless of the direction of this feedback (i.e., the drinking norm set). Furthermore, younger adults reported fewer drinking days, but more binge drinking and more alcohol use–related problems, as well as higher explicit social attunement. Explicit social attunement was specifically associated with higher alcohol use and related problems (AUDIT scores) rather than metrics of heaviness of alcohol use only (TLFB outcomes). Explicit social attunement was only associated with implicit social attunement to peer feedback indicative of higher willingness to drink in the SND settings and observed correlation was small (*r* = 0.12), indicating that implicit and explicit social attunement are likely different social processes. In line with these differences, explicit social attunement—but none of the implicit social attunement measures—mediated the association between age and alcohol use and related problems (AUDIT scores). These effects were guided by those with AUDIT scores above the cut-off for problematic alcohol use (AUDIT > 7). Also, in line with the lack of effects found for any of the TLFB outcomes, the effect was guided by higher scores on the AUDIT items indicating problematic use (AUDIT-P; items 4–10) rather than the items indicating heaviness of use (AUDIT-C; items 1–3). Alternatively, the mediating role of explicit, but not implicit social attunement, in the relation between age and problematic alcohol use may in part be explained by social conformity behavior, which is likely to be more closely related to the explicit than implicit outcomes. It is recommended that future studies include more measures to allow differentiation between explicit social attunement and conformity. Although the mediation analyses should be considered exploratory, the results indicate that the association between age and alcohol use and related problems might be partially guided by increased explicit social attunement in younger individuals. Longitudinal studies—assessing the change of these associations during the transition from adolescence to adulthood and beyond—are crucial to confirm these associations.

The new implicit social attunement task showed a couple of clear strengths. The task induced implicit social attunement in the direction of the peer feedback. Furthermore, the use of fictional peers of a similar age prevented potential selection effects. Mundt et al. (2012) showed that individuals are known to select peers that they relate to in terms of behavior and interests, potentially resulting in peer feedback biased towards one’s own behavior/opinion within those peer groups. To experimentally assess the complexity of peer influence more generally, the stimuli can also easily be adapted to include other drugs (e.g., cannabis) or other social behaviors to assess willingness to participate in those behaviors based on implicit norms presented across conditions (e.g., adding different social settings and/or type of drink, food, or behavior) and whether this willingness changes depending on the provided feedback (e.g., same, higher, or lower).

However, some limitations must be noted. This study is fully cross-sectional and longitudinal studies are needed to confirm the temporal precedence of the observed associations. Furthermore, the fictional nature of the peers and the relative emotional distance to this peer group affects ecological validity. Future research could gather information on one’s social network, providing personal peer group feedback, and consider the drinking norms in one’s social circle in the analysis. Also, due to the response-dependent probabilistic feedback, individuals reporting very low or very high willingness across trials received limited feedback in one of the directions. While general implicit social attunement (regardless of feedback direction) can always be assessed, separating positive and negative influence might not always be an option. Furthermore, results showed remarkable inconsistencies between alcohol use and related problems—as assessed with the AUDIT—and self-reported recent alcohol use—as assess with the TLFB. While it could well be that the effects found are indeed primarily associated with alcohol use–related problems rather than heaviness of recent use, the TLFB results could also be affected by the relatively low variance in the past 2-week drinking with most participants reporting drinking less than 30 drinks, and by age differences in binge drinking. Exploratory assessments revealed that binge drinking was negatively associated with age (*r* =  − 0.113, *p* = 0.014) and while regular binge drinking has been associated with higher AUDIT scores it might result in underestimation of use on the TLFB (Collins et al., 2008). Also, while the inclusion of participants from a broad age range is a clear strength of this study, we did not manage to recruit an equal number of participants across ages, resulting in an overrepresentation of participants under forty. Replication including a larger number of older adults is warranted. Moreover, research including heavier alcohol users is needed to assess the generalizability to hazardous users as most AUDIT scores were below the at-risk cut-off (Saunders et al., 1993) and last 2-week alcohol use indicated less than daily use (Table 1). Furthermore, studies are needed to assess associations between implicit social attunement and other types of social learning to evaluate divergent validity of the task.

This study shows the potential of using the implicit social attunement task in unravelling the complex effects of social settings and peer feedback on alcohol consumption across age ranges. Results indicated that peer influence might act as protective or risk factor for alcohol use depending on the social setting and highlights the potential differences between implicit and explicit social attunement behaviors in their associations with age and alcohol use and related problems.

## Supplementary Information

Below is the link to the electronic supplementary material.

Supplementary file1 (PDF 439 KB)

## Data Availability

The data that support the findings of this study are available from the corresponding author upon reasonable request.

## Declarations

### Preregistration

This project was not preregistered.

### Preprint

A preprint version of this manuscript has been uploaded to PsyArXiv (10.31234/osf.io/zx7ky).
