# Supplementary material for: Implicit Social Attunement and Alcohol Use: The Effect of Peer Feedback on Willingness to Drink in Social Settings
Source: Int J Ment Health Addict. 2024 Aug 12;23(6):4584–99. doi: 10.1007/s11469-024-01371-4 (PMC12698825; doi:10.1007/s11469-024-01371-4)
Supplement: Supplementary file 1 — Supplementary file1 (PDF 439 KB) [file 11469_2024_1371_MOESM1_ESM.pdf]

SUPPLEMENTARY MATERIALS

Implicit Social Attunement & Alcohol Use: The Effect of Peer Feedback on Willingness to Drink in Social Settings

dr. Emese Kroon *[1,2], Ran Zhang MSc.[2], Karis Colyer-Patel MSc.[2], Alix Weidema MSc.[2], Doğa Ünsal MSc.[2], dr. Helle Larsen[1], dr. Janna Cousijn[1,2]

[1] Department of Psychology, University of Amsterdam, the Netherlands

[2] Neuroscience of Addiction (NofA) Lab, Center for Substance Use and Addiction Research (CESAR), Department of Psychology, Education & Child Studies, Erasmus University Rotterdam, The Netherlands

*Correspondence: Emese Kroon, P.O. box 15916, 1001 NK Amsterdam, The Netherlands, emesekroon@gmail.com, +31 10 754 6133

Declarations of interest: none

Funding: This research was supported by the ERC-ST grant 947761 awarded to Janna Cousijn.

Data Accessibility: The data that support the findings of this study are available from the corresponding author upon reasonable request.

Preregistration: This project was not preregistered.

Table S1. Implicit Social Attunement Task

Outcome	N	M(SD)	Range	One-Sample t-test
**Willingness to drink[a]**				
Social Alcohol Drink (SAD)	566	5.70 (2.68)	1-10	-
Social Non-Alcohol Drink (SNAD)	566	4.03 (2.20)	1-10	-
Social Non-Drink (SND)	566	2.02 (1.30)	1-10	-
**Change in willingness to drink**				
SAD – high	542	.18(1.02)	-3.75-6.50	t(541) = 4.16, *p* <.001
SAD – low	517	-.55(1.02)	-5.56-6.33	t(516) = -12.2, *p* <.001
SAD – same	566	-.07(1.31)	-6.50-6.00	t(565) = -1.29, *p* =.199
SNAD – high	564	.24(.92)	-2.75-4.00	t(563) = 6.06, *p* <.001
SNAD – low	490	-.69(1.19)	-5.00-3.25	t(489) = -12.9, *p* <.001
SNAD – same	566	-.08(1.22)	-5.00-5.00	t(565) = -1.47, *p* = .143
SND – high	564	.16(.73)	-1.63-5.13	t(563) = 5.15, *p* <.001
SND – low	359	-1.02(1.63)	-9.00-4.80	t(358) = -11.8, *p* <.001
SND - same	566	.04(.92)	-3.50-5.50	t(565) = 1.14, *p* = .254
**Implicit social attunement (ISA)[b] – per setting**				
SAD ISA+	484	0.27 (1.33)	-5.71-6.5	t(541) = 4.52, *p* <.001
SAD ISA-	459	0.63 (2.04)	-11.33-11.06	t(516) = 7.03, *p* <.001
SNAD ISA+	504	0.33 (1.30)	-5.25-6.63	t(563) = 5.65, *p* <.001
SNAD ISA-	438	0.85 (2.13)	-6.17-7.5	t(489) = 8.13, *p* <.001
SND ISA+	504	0.11 (0.81)	-4.06-3.75	t(563) = 3.18, *p* = .002
SND ISA-	322	0.98 (2.20)	-9.8-9	t(358) = 8.30, *p* <.001

Note. [a] scale from 1-10. [b] Scores all positive, reflecting magnitude of ISA in the described direction. SAD: social alcohol drink, SNAD: social non-alcohol drink, SND: social non-drink, high: peer feedback indicating higher willingness to drink, low: peer feedback indicating lower willingness to drink, same: peer feedback identical to own willingness to drink; ISA+: implicit social attunement to peer feedback indicating higher willingness to drink, controlled for same feedback trials responses. ISA-: implicit social attunement to peer feedback indicating lower willingness to drink, controlled for same feedback trials responses.

Table S2. Holm corrected post-hoc analysis assessing the interaction of Social Setting and Feedback Type in their effect on ISA scores

**Comparison**		**Mean difference**	**SE**	**95% CI**	**t**	**df**	**p_{holm}**	**d**
SAD+	SNAD+	-0.037	0.127	-.410:.336	-0.294	325	1.000	.033
SAD+	SND+	0.143	0.127	-.230:.516	1.127	325	0.780	.125
SNAD+	SND+	0.180	0.127	-.193:.553	1.421	325	1.000	.158
SAD-	SNAD-	-0.033	0.127	-.406:.340	-0.260	325	1.000	.029
SAD-	SND-	-0.257	0.127	-.630:.116	-2.024	325	0.302	.225
SNAD-	SND-	-0.224	0.127	-.597:.149	-1.764	325	0.390	.196
SAD+	SAD-	-0.305	0.104	-.612:.002	-2.929	325	**0.035**	.325
SAD+	SNAD-	-0.338	0.138	-.745:.068	-2.449	325	0.116	.272
SAD+	SND-	-0.562	0.138	-.969:-.156	-4.068	325	**<.001**	.451
SNAD+	SAD-	-0.268	0.138	-.674:.138	-1.940	325	0.316	.215
SNAD+	SNAD-	-0.301	0.104	-.608:.006	-2.888	325	**0.036**	.320
SNAD+	SND-	-0.525	0.138	-.931:-.118	-3.798	325	**0.002**	.421
SND+	SAD-	-0.448	0.138	-.855:-.042	-3.244	325	**0.013**	.360
SND+	SNAD-	-0.481	0.138	-.888:-.075	-3.483	325	**0.006**	.386
SND+	SND-	-0.705	0.104	-1.012:-.398	-6.764	325	**<.001**	.750

Note. SAD: social alcohol drink, SNAD: social non-alcohol drink, SND: social non-drink, +: peer feedback indicating higher willingness to drink, -: peer feedback indicating lower willingness to drink. All ISA scores (+ and -) are corrected for change on same feedback trials.

Table S3. Full overview of the Spearman correlations between implicit social attunement, explicit social attunement, age, and alcohol use

Measures			1	2	3	4	5	6	7	8	9	10	11	12
1	SAD ISA+	*r*	-	-	-	-	-	-	-	-	-	-	-	-
		p	-	-	-	-	-	-	-	-	-	-	-	-
2	SAD ISA-	*r*	**0.440**	-	-	-	-	-	-	-	-	-	-	-
		p	**<.001**	-	-	-	-	-	-	-	-	-	-	-
3	SNAD ISA+	*r*	0.004	0.004	-	-	-	-	-	-	-	-	-	-
		p	0.925	0.930	-	-	-	-	-	-	-	-	-	-
4	SNAD ISA-	*r*	**-0.121**	**0.318**	**0.496**	-	-	-	-	-	-	-	-	-
		p	**0.013**	**<.001**	**<.001**	-	-	-	-	-	-	-	-	-
5	SND ISA+	*r*	**0.165**	-0.066	-0.018	-0.158	-	-	-	-	-	-	-	-
		p	**<.001**	0.159	0.682	<.001	-	-	-	-	-	-	-	-
6	SND ISA-	*r*	0.007	0.025	**0.146**	**0.350**	**0.300**	-	-	-	-	-	-	-
		p	0.907	0.660	**0.009**	**<.001**	**<.001**	-	-	-	-	-	-	-
7	SAQ	*r*	0.046	-0.066	0.019	-0.065	**0.118**	-0.010	-	-	-	-	-	-
		p	0.314	0.158	0.669	0.174	**0.008**	0.853	-	-	-	-	-	-
8	Age	*r*	-0.058	-0.029	**-0.168**	**-0.143**	-0.002	0.006	**-0.161**	-	-	-	-	-
		p	0.204	0.541	**<.001**	**0.003**	0.965	0.909	**<.001**	-	-	-	-	-
9	TLFB – Standard drinks	*r*	**0.111**	-0.061	0.017	-0.064	-0.021	**-0.142**	-0.003	0.000	-	-	-	-
		p	**0.015**	0.192	0.695	0.184	0.632	**0.011**	0.947	0.991	-	-	-	-
10	TLFB – Drinking days	*r*	**0.113**	-0.066	0.004	-0.031	-0.043	-0.073	-0.036	**0.119**	**0.898**	-	-	-
		p	**0.013**	0.155	0.933	0.524	0.330	0.191	0.414	**0.007**	**<.001**	-	-	-
11	TLFB – Binge days	*r*	0.063	-0.057	0.016	**-0.094**	-0.032	**-0.155**	0.041	**-0.165**	**0.772**	**0.521**	-	-
		p	0.167	0.223	0.717	**0.049**	0.480	**0.005**	0.353	**<.001**	**<.001**	**<.001**	-	-
12	AUDIT	*r*	**0.147**	0.014	**0.113**	-0.053	0.010	**-0.175**	**0.142**	**-0.173**	**0.660**	**0.532**	**0.606**	-
		p	**0.001**	0.770	**0.011**	0.270	0.830	**0.002**	**0.001**	**<.001**	**<.001**	**<.001**	**<.001**	-

Note. SAD: social alcohol drink, SNAD: social non-alcohol drink, SND: social non-drink, ISA+: implicit social attunement to peer feedback indicating higher willingness to drink, controlled for same feedback trials responses. ISA-: implicit social attunement to peer feedback indicating lower willingness to drink, controlled for same feedback trials responses. AUDIT: alcohol use disorder identification test, TLFB: timeline follow-back. SAQ: social attunement questionnaire scores of explicit social attunement.

**Table S4. Mediation results AUDIT**					
**Model**	**Results**				
**SAQ**	***B***	***SE* (B)**	**95% *CI***	***z***	***p***
AUDIT ~ Age (c)	-.076	.021	-.115:-.039	-3.620	**<.001**
SAQ ~ Age (a)	-.156	.038	-.229:-.083	-4.107	**<.001**
AUDIT ~ SAQ (b)	.082	.024	.039:.139	3.407	**<.001**
Indirect (ab)	-.013	.005	-.027:-.006	-2.622	**.009**
Direct (c`)	-.076	.021	-.115:-.039	-3.620	**<.001**
Total (ab + c`)	-.088	.021	-.128:-.052	-4.253	**<.001**
**SAD ISA+**	***B***	***SE* (B)**	**95%*CI***	***z***	***p***
AUDIT ~ Age (c)	-.086	.021	-.123:-.049	-4.126	**<.001**
SAD ISA+ ~ Age (a)	-.008	.005	-.016:-.000	-1.535	.125
AUDIT ~ SAD ISA+ (b)	.345	.193	-.183:.0781	1.791	.073
Indirect (ab)	-.003	.002	-.011:.001	-1.164	.244
Direct (c`)	-.086	.021	-.123:-.049	-4.126	**<.001**
Total (ab + c`)	-.088	.021	-.124:-.051	-4.253	**<.001**
**SAD ISA-**	***B***	***SE* (B)**	**95% *CI***	***z***	***p***
AUDIT ~ Age (c)	-.088	.021	-.123:-.048	-4.243	**<.001**
SAD ISA- ~ Age (a)	-.008	.008	-.023:.007	-.997	.319
AUDIT ~ SAD ISA- (b)	.012	.129	-.259:.237	.094	.925
Indirect (ab)	.000	.001	-.004:.002	-.094	.925
Direct (c`)	-.088	.021	-.123:-.048	-4.243	**<.001**
Total (ab + c`)	-.088	.021	-.123:-.048	-4.253	**<.001**
**SNAD ISA+**	***B***	***SE* (B)**	**95% *CI***	***z***	***p***
AUDIT ~ Age (c)	-.083	.021	-.121:-.044	-3.984	**<.001**
SNAD ISA+ ~ Age (a)	-.016	.005	-.026:-.008	-3.343	**<.001**
AUDIT ~ SNAD ISA+ (b)	.299	.194	-.244:.829	1.543	.829
Indirect (ab)	-.005	.003	-.016:.003	-1.408	.159
Direct (c`)	-.083	.021	-.121:-.044	-3.984	**<.001**
Total (ab + c`)	-.088	.021	-.127:-.050	-4.253	**<.001**
**SNAD ISA-**	***B***	***SE* (B)**	**95% *CI***	***z***	***p***
AUDIT ~ Age (c)	-.091	.021	-.125:-.054	-4.345	**<.001**
SNAD ISA- ~ Age (a)	-.019	.009	-.038:-.002	-2.104	**.035**
AUDIT ~ SNAD ISA- (b)	-.127	.126	-.387:.146	-.1013	.311
Indirect (ab)	.002	.003	-.002:.011	.917	.359
Direct (c`)	-.091	.021	-.125:-.054	-4.345	**<.001**
Total (ab + c`)	-.088	.021	-.124:-.050	-4.253	**<.001**
**SND ISA+**	***B***	***SE* (B)**	**95% *CI***	***z***	***p***
AUDIT ~ Age (c)	-.088	.021	-.130:-.048	-4.253	**<.001**
SND ISA+ ~ Age (a)	-.000	.003	-.005:.004	-.217	.828
AUDIT ~ SND ISA+ (b)	-.004	.307	-.911:.837	-.013	.990
Indirect (ab)	.000	.000	-.002:.003	.013	.990
Direct (c`)	-.088	.021	-.130:-.048	-4.253	**<.001**
Total (ab + c`)	-.088	.021	-.129:-.048	-4.253	**<.001**
**SND ISA-**	***B***	***SE* (B)**	**95% *CI***	***z***	***p***
AUDIT ~ Age (c)	-.082	.021	-.116:-.040	-3.938	**<.001**
SND ISA- ~ Age (a)	.013	.013	-.010:.036	1.201	.230
AUDIT ~ SND ISA- (b)	-.501	.134	-.768:-.206	-3.730	**<.001**
Indirect (ab)	-.007	.006	-.022:.004	-1.149	.251
Direct (c`)	-.082	.021	-.116:-.040	-3.938	**<.001**
Total (ab + c`)	-.088	.021	-.122:-.048	-4.253	**<.001**

Note: Maximum likelihood estimation used in all models. Bootstrap = 1000; SE: standard error; SAD: social alcohol drink, SNAD: social non-alcohol drink, SND: social non-drink, ISA+: implicit social attunement to peer feedback indicating higher willingness to drink, controlled for same feedback trials responses. ISA-: implicit social attunement to peer feedback indicating lower willingness to drink, controlled for same feedback trials responses. AUDIT: alcohol use disorder identification test, SAQ: social attunement questionnaire scores of explicit social attunement.

Table S5. Mediation results TLFB – Standard Drinks

Model	Results				
**SAQ**	***B***	***SE* (B)**	**95% *CI***	***z***	***p***
TLFB ~ Age (c)	.012	.065	-.110:.150	.185	.853
SAQ ~ Age (a)	-.156	.038	-.229:-.076	-4.107	**<.001**
TLFB ~ SAQ (b)	.038	.075	-.084:.166	.514	.608
Indirect (ab)	-.006	.012	-.028:.012	-.510	.610
Direct (c`)	.012	.065	-.110:.150	.185	.853
Total (ab + c`)	.006	.064	-.110:.148	.094	.925
**SAD ISA+**	***B***	***SE* (B)**	**95% *CI***	***z***	***p***
TLFB ~ Age (c)	.011	.064	-.112:.144	.167	.867
SAD ISA+ ~ Age (a)	-.008	.005	-.017:.000	-1.563	**<.001**
TLFB ~ SAD ISA+ (b)	.590	.602	-.889:2.076	.980	.327
Indirect (ab)	-.005	.006	-.027L.005	-.819	.413
Direct (c`)	.011	.064	-.112:.144	.167	.867
Total (ab + c`)	.006	.064	-.121:1.43	.094	.925
**SAD ISA-**	***B***	***SE* (B)**	**95% *CI***	***z***	***p***
TLFB ~ Age (c)	.004	.064	-.122:.146	.063	.950
SAD ISA- ~ Age (a)	-.008	.008	-.022:.006	-.989	.323
TLFB ~ SAD ISA- (b)	-.247	.381	-1.167:.666	-.648	.517
Indirect (ab)	.002	.004	-.004:.023	.545	.586
Direct (c`)	.004	.064	-.122:.146	.063	.950
Total (ab + c`)	.006	.064	-.121:.150	.094	.925
**SNAD ISA+**	***B***	***SE* (B)**	**95% *CI***	***z***	***p***
TLFB ~ Age (c)	.004	.064	-.119:.149	.063	.950
SNAD ISA+ ~ Age (a)	-.016	.005	-.026:-.008	-3.362	**<.001**
TLFB ~ SNAD ISA+ (b)	-.118	.587	-1.503:1.203	-.201	.841
Indirect (ab)	.002	.010	-.018:.029	.201	.841
Direct (c`)	.004	.064	-.119:.149	.063	.950
Total (ab + c`)	.006	.064	-.112:.152	.094	.925
**SNAD ISA-**	***B***	***SE* (B)**	**95% *CI***	***z***	***p***
TLFB ~ Age (c)	-.003	.064	-.130:.149	-.049	.961
SNAD ISA- ~ Age (a)	-.019	.009	-.036:-.002	-2.078	**.038**
TLFB ~ SNAD ISA- (b)	-.491	.369	-1.226:.246	-1.332	.183
Indirect (ab)	.009	.008	-.002:.035	1.136	.256
Direct (c`)	-.003	.064	-.130:.149	-.049	.961
Total (ab + c`)	.006	.064	-.119:.162	.094	.925
**SND ISA+**	***B***	***SE* (B)**	**95% *CI***	***z***	***p***
TLFB ~ Age (c)	.005	.064	-.111:.145	.083	.934
SND ISA+ ~ Age (a)	.001	.003	-.005:.004	-.210	.834
TLFB ~ SND ISA+ (b)	-1.154	.928	-3.500:.953	-1.244	.213
Indirect (ab)	.001	.004	-.005:.012	.207	.836
Direct (c`)	.005	.064	-.111:.145	.083	.934
Total (ab + c`)	.006	.064	-.110:.146	.094	.925
**SND ISA-**	***B***	***SE* (B)**	**95% *CI***	***z***	***p***
TLFB ~ Age (c)	.020	.064	-.105:.165	.317	.751
SND ISA- ~ Age (a)	.014	.011	-.010:.038	1.232	.218
TLFB ~ SND ISA- (b)	-1.048	.394	-1.900:-.146	-2.660	**.008**
Indirect (ab)	-.014	.013	-.056:.006	-1.123	.261
Direct (c`)	.020	.064	-.105:.165	.317	.751
Total (ab + c`)	.006	.064	-.121:.146	.094	.925

Note: Maximum likelihood estimation used in all models. Bootstrap = 1000; SE: standard error; SAD: social alcohol drink, SNAD: social non-alcohol drink, SND: social non-drink, ISA+: implicit social attunement to peer feedback indicating higher willingness to drink, controlled for same feedback trials responses. ISA-: implicit social attunement to peer feedback indicating lower willingness to drink, controlled for same feedback trials responses. TLFB: timeline follow-back. SAQ: social attunement questionnaire scores of explicit social attunement.

Table S6. Mediation results TLFB – Drinking Days

Model	Results				
**SAQ**	***B***	***SE* (B)**	**95% *CI***	***z***	***p***
TLFB ~ Age (c)	.050	.014	.019:.080	3.590	**<.001**
SAQ ~ Age (a)	-.120	.032	-.186:-.051	-3.763	**<.001**
TLFB ~ SAQ (b)	-.010	.019	-.052:.034	-.538	.591
Indirect (ab)	.001	.002	-.004:.007	.533	.594
Direct (c`)	.050	.014	.019:.080	3.590	**<.001**
Total (ab + c`)	.051	.014	.022:.082	3.728	**<.001**
**SAD ISA+**	***B***	***SE* (B)**	**95% *CI***	***z***	***p***
TLFB ~ Age (c)	.053	.014	.023:.084	3.873	**<.001**
SAD ISA+ ~ Age (a)	-.008	.005	-.016:.000	-1.627	.104
TLFB ~ SAD ISA+ (b)	.237	.127	-.029:.524	1.868	.062
Indirect (ab)	-.002	.002	-.007:.000	-1.209	.227
Direct (c`)	.053	.014	.023:.084	3.873	**<.001**
Total (ab + c`)	.051	.014	.021:.083	3.728	**<.001**
**SAD ISA-**	***B***	***SE* (B)**	**95% *CI***	***z***	***p***
TLFB ~ Age (c)	.051	.014	.024:.084	3.728	**<.001**
SAD ISA- ~ Age (a)	-.009	.008	-.023:.006	-1.111	.267
TLFB ~ SAD ISA- (b)	.018	.084	-.190:,229	.216	.829
Indirect (ab)	.000	.000	-.004:.002	-.211	.833
Direct (c`)	.051	.014	.023:.084	3.735	**<.001**
Total (ab + c`)	.051	.014	.024:.084	3.728	**<.001**
**SNAD ISA+**	***B***	***SE* (B)**	**95% *CI***	***z***	***p***
TLFB ~ Age (c)	.051	.014	.024:.083	3.728	**<.001**
SNAD ISA+ ~ Age (a)	-.017	.005	-.026:-.008	-3.565	**<.001**
TLFB ~ SNAD ISA+ (b)	-.020	.127	-.310:.251	-.161	.872
Indirect (ab)	.000	.002	-.004:.006	.160	.873
Direct (c`)	.051	.014	.024:.081	3.657	**<.001**
Total (ab + c`)	.051	.014	.024:.083	3.728	**<.001**
**SNAD ISA-**	***B***	***SE* (B)**	**95% *CI***	***z***	***p***
TLFB ~ Age (c)	.050	.014	.024:.079	3.647	**<.001**
SNAD ISA- ~ Age (a)	-.020	.009	-.038:-.003	-2.257	**.024**
TLFB ~ SNAD ISA- (b)	-.038	.082	-.216:.159	-.460	.645
Indirect (ab)	.000	.002	-.003:.006	.457	.648
Direct (c`)	.050	.014	.024:.079	3.647	**<.001**
Total (ab + c`)	.051	.014	.024:.081	3.728	**<.001**
**SND ISA+**	***B***	***SE* (B)**	**95% *CI***	***z***	***p***
TLFB ~ Age (c)	.051	.014	.019:.081	3.720	**<.001**
SND ISA+ ~ Age (a)	-.000	.003	-.005:.004	-.251	.802
TLFB ~ SND ISA+ (b)	-.262	.200	-.731:.147	-1.308	.191
Indirect (ab)	.000	.000	-.000:.003	.247	.805
Direct (c`)	.051	.014	.019:.081	3.720	**<.001**
Total (ab + c`)	.051	.014	.018:.081	3.728	**<.001**
**SND ISA-**	***B***	***SE* (B)**	**95% *CI***	***z***	***p***
TLFB ~ Age (c)	.052	.014	.024:.081	3.809	**<.001**
SND ISA- ~ Age (a)	.014	.011	-.008:.038	1.263	.206
TLFB ~ SND ISA- (b)	-.096	.094	-.300:.130	-1.019	.308
Indirect (ab)	-.001	.002	-.010:.001	-.790	.430
Direct (c`)	.052	.014	.024:.081	3.809	**<.001**
Total (ab + c`)	.051	.014	.022:.080	3.728	**<.001**

Note: Maximum likelihood estimation used in all models. Bootstrap = 1000; SE: standard error; SAD: social alcohol drink, SNAD: social non-alcohol drink, SND: social non-drink, ISA+: implicit social attunement to peer feedback indicating higher willingness to drink, controlled for same feedback trials responses. ISA-: implicit social attunement to peer feedback indicating lower willingness to drink, controlled for same feedback trials responses. TLFB: timeline follow-back. SAQ: social attunement questionnaire scores of explicit social attunement.

**Table S7. Mediation results TLFB – Binge Days**					
**Model**	**Results**				
**SAQ**	***B***	***SE* (B)**	**95% *CI***	***z***	***p***
TLFB ~ Age (c)	-.014	.007	-.024:.000	-2.072	**.038**
SAQ ~ Age (a)	-.120	.032	-.190:-.056	-3.763	**<.001**
TLFB ~ SAQ (b)	-.004	.009	-.026:.011	-.433	.665
Indirect (ab)	.000	.001	-.001:.004	.430	.667
Direct (c`)	-.014	.007	-.024:.000	-2.072	**.038**
Total (ab + c`)	-.013	.007	-.024:.002	-2.030	**.042**
**SAD ISA+**	***B***	***SE* (B)**	**95% *CI***	***z***	***p***
TLFB ~ Age (c)	-.013	.007	-.024:.004	-1.952	.051
SAD ISA+ ~ Age (a)	-.008	.005	-.016:.000	-1.592	.111
TLFB ~ SAD ISA+ (b)	.060	.060	-.097:.251	1.001	.317
Indirect (ab)	-.000	-.000	-.003:.000	-.840	.401
Direct (c`)	-.013	.007	-.024:.004	-1.952	.051
Total (ab + c`)	-.013	.007	-.024:.004	-2.030	**.042**
**SAD ISA-**	***B***	***SE* (B)**	**95% *CI***	***z***	***p***
TLFB ~ Age (c)	-.013	.007	-.024:.003	-2.030	**.042**
SAD ISA- ~ Age (a)	-.009	.008	-.023:.006	-1.104	.270
TLFB ~ SAD ISA- (b)	-.007	.039	-.109:.137	-.180	.857
Indirect (ab)	-.000	-.000	-.001:.002	-.178	.859
Direct (c`)	-.013	.007	-.024:.003	-2.037	**.042**
Total (ab + c`)	-.013	.007	-.024:.003	-2.030	**.042**
**SNAD ISA+**	***B***	***SE* (B)**	**95% *CI***	***z***	***p***
TLFB ~ Age (c)	-.014	.007	-.025:.002	-2.114	**.035**
SNAD ISA+ ~ Age (a)	-.017	.005	-.026:-.008	-3.564	**<.001**
TLFB ~ SNAD ISA+ (b)	-.042	.060	-.161:.083	-.687	.492
Indirect (ab)	.000	.001	-.001:.003	.675	.500
Direct (c`)	-.014	.007	-.025:.002	-2.114	**.035**
Total (ab + c`)	-.013	.007	-.024:.002	-2.030	**.042**
**SNAD ISA-**	***B***	***SE* (B)**	**95% *CI***	***z***	***p***
TLFB ~ Age (c)	-.014	.007	-.025:-.000	-2.202	**.028**
SNAD ISA- ~ Age (a)	-.020	.009	-.041:.006	-2.294	**.022**
TLFB ~ SNAD ISA- (b)	-.059	.037	-.135:.024	-1.573	.116
Indirect (ab)	.001	.000	.000:.004	1.298	.194
Direct (c`)	-.014	.007	-.025:-.000	-2.202	**.028**
Total (ab + c`)	-.013	.007	-.024:.000	-2.030	**.042**
**SND ISA+**	***B***	***SE* (B)**	**95% *CI***	***z***	***p***
TLFB ~ Age (c)	-.013	.007	-.024:.002	-2.032	**.042**
SND ISA+ ~ Age (a)	-.000	.003	-.006:.004	-.263	.793
TLFB ~ SND ISA+ (b)	-.020	.095	-.216:.174	-.215	.830
Indirect (ab)	.000	.000	-.000:.000	.166	.868
Direct (c`)	-.013	.007	-.024:.002	-2.032	**.042**
Total (ab + c`)	-.013	.007	-.024:.002	-2.030	**.042**
**SND ISA-**	***B***	***SE* (B)**	**95% *CI***	***z***	***p***
TLFB ~ Age (c)	-.012	.007	-.023:.004	-1.852	.064
SND ISA- ~ Age (a)	.013	.011	-.007:.035	1.210	.226
TLFB ~ SND ISA- (b)	-.086	.041	-.199:.015	-2.078	**.038**
Indirect (ab)	-.001	.001	-.005:.000	-1.056	.291
Direct (c`)	-.012	.007	-.023:.004	-1.852	.064
Total (ab + c`)	-.013	.007	-.024:.003	-2.030	**.024**

Note: Maximum likelihood estimation used in all models. Bootstrap = 1000; SE: standard error; SAD: social alcohol drink, SNAD: social non-alcohol drink, SND: social non-drink, ISA+: implicit social attunement to peer feedback indicating higher willingness to drink, controlled for same feedback trials responses. ISA-: implicit social attunement to peer feedback indicating lower willingness to drink, controlled for same feedback trials responses. TLFB: timeline follow-back. SAQ: social attunement questionnaire scores of explicit social attunement.

Table S8. Sensitivity analysis: mediation results SAQ in high and low risk drinkers split on AUDIT score

Model	Results				
**AUDIT > 7 – High risk**	***B***	***SE* (B)**	**95% *CI***	***z***	***p***
AUDIT ~ Age (c)	.008	.036	-.069:.078	.216	.829
SAQ ~ Age (a)	-.120	.032	-.190:-.058	-3.763	**<.001**
AUDIT ~ SAQ (b)	.104	.040	.026:.181	2.605	**.009**
Indirect (ab)	-.013	.006	-.029:-.005	-2.142	**.032**
Direct (c`)	.008	.036	-.069:.078	.216	.829
Total (ab + c`)	-.005	.037	-.079:.063	-.127	.899
**AUDIT < 8 – Low risk**	***B***	***SE* (B)**	**95%*CI***	***z***	***p***
AUDIT ~ Age (c)	-.006	.020	-.024:.015	-.561	.575
SAQ ~ Age (a)	-.120	.032	-.180:-.051	-3.763	**<.001**
AUDIT ~ SAQ (b)	.015	.015	-.014:.044	.992	.321
Indirect (ab)	-.002	.002	-.006:.001	-.960	.337
Direct (c`)	-.006	.020	-.024:.015	-.561	.575
Total (ab + c`)	-.007	.010	-.025:.014	-.750	.453

Note: Maximum likelihood estimation used in all models. Bootstrap = 1000; SE: standard error; AUDIT: alcohol use disorder identification test, SAQ: social attunement questionnaire scores of explicit social attunement.

Table S9. Sensitivity analysis: Mediation results SAQ using AUDIT-C and AUDIT-P

Model	Results				
**AUDIT-C**	***B***	***SE* (B)**	**95% *CI***	***z***	***p***
AUDIT-C ~ Age (c)	-.024	.009	-.043:-.006	-2.558	**.011**
SAQ ~ Age (a)	-.120	.032	-.187:-.057	-3.763	**<.001**
AUDIT-C ~ SAQ (b)	.023	.023	-.002:.050	1.786	.074
Indirect (ab)	-.003	.003	-.007:.000	-1.613	.107
Direct (c`)	-.024	.009	-.043:-.006	-2.558	**.011**
Total (ab + c`)	-.026	.009	-.046:-.009	-2.883	**.004**
**AUDIT-P**	***B***	***SE* (B)**	**95%*CI***	***z***	***p***
AUDIT-P ~ Age (c)	-.050	.014	-.076:-.024	-3.608	**<.001**
SAQ ~ Age (a)	-.120	.032	-.187:-.054	-3.763	**<.001**
AUDIT-P ~ SAQ (b)	.070	.019	.032:.110	3.709	**<.001**
Indirect (ab)	-.008	.003	-.015:-.004	-2.641	**.008**
Direct (c`)	-.050	.014	-.076:-.024	-3.608	**<.001**
Total (ab + c`)	-.058	.014	-.083:-.033	-4.222	**<.001**

Note: Maximum likelihood estimation used in all models. Bootstrap = 1000; SE: standard error; AUDIT: alcohol use disorder identification test, SAQ: social attunement questionnaire scores of explicit social attunement.
